# Fluorescent Probes for Mammalian Thioredoxin Reductase: Mechanistic Analysis, Construction Strategies, and Future Perspectives

**DOI:** 10.3390/bios13080811

**Published:** 2023-08-13

**Authors:** Zilong Song, Chengwu Fan, Jintao Zhao, Lei Wang, Dongzhu Duan, Tong Shen, Xinming Li

**Affiliations:** 1 Natural Medicine Research & Development Center, Lanzhou Jiaotong University, Lanzhou 730070, China; songzl16@lzu.edu.cn (Z.S.); cwufan@163.com (C.F.); 11200606@stu.lzjtu.edu.cn (L.W.); 2 School of Chemistry and Chemical Engineering, Nanjing University of Science & Technology, Nanjing 210094, China; zhaojt2013@lzu.edu.cn (J.Z.); lixmchem@njust.edu.cn (X.L.); 3 Shaanxi Key Laboratory of Phytochemistry, College of Chemistry and Chemical Engineering, Baoji University of Arts and Sciences, Baoji 721013, China; duandongzhu@126.com

**Keywords:** fluorescent probe, redox chemistry, thioredoxin reductase, thioredoxin, 1,2-dithiolane, selenenylsulfide, diselenide, disulfide

## Abstract

The modulation of numerous signaling pathways is orchestrated by redox regulation of cellular environments. Maintaining dynamic redox homeostasis is of utmost importance for human health, given the common occurrence of altered redox status in various pathological conditions. The cardinal component of the thioredoxin system, mammalian thioredoxin reductase (TrxR) plays a vital role in supporting various physiological functions; however, its malfunction, disrupting redox balance, is intimately associated with the pathogenesis of multiple diseases. Accordingly, the dynamic monitoring of TrxR of live organisms represents a powerful direction to facilitate the comprehensive understanding and exploration of the profound significance of redox biology in cellular processes. A number of classic assays have been developed for the determination of TrxR activity in biological samples, yet their application is constrained when exploring the real-time dynamics of TrxR activity in live organisms. Fluorescent probes offer several advantages for in situ imaging and the quantification of biological targets, such as non-destructiveness, real-time analysis, and high spatiotemporal resolution. These benefits facilitate the transition from a poise to a flux understanding of cellular targets, further advancing scientific studies in related fields. This review aims to introduce the progress in the development and application of TrxR fluorescent probes in the past years, and it mainly focuses on analyzing their reaction mechanisms, construction strategies, and potential drawbacks. Finally, this study discusses the critical challenges and issues encountered during the development of selective TrxR probes and proposes future directions for their advancement. We anticipate the comprehensive analysis of the present TrxR probes will offer some glitters of enlightenment, and we also expect that this review may shed light on the design and development of novel TrxR probes.

## 1. Introduction 

The thioredoxin system is generally considered to comprise thioredoxin reductase (TrxR), thioredoxin (Trx), and nicotinamide adenine dinucleotide phosphate (NADPH) [1,2]. This system is a ubiquitous disulfide oxidoreductase antioxidant network, and it preserves highly evolutionary conservation throughout all living organisms, which highlights its significance in biological systems [3,4]. It is well appreciated that the Trx system plays pivotal roles in maintaining intracellular redox homeostasis and exerts multivalent physiological functions via regulating numerous redox-associated signaling pathways [5]. An abnormal gain or loss of functions of the system is tightly linked to various pathological conditions, such as cancer [6,7,8], neurodegenerative disorders [9], and cardiovascular diseases [10].

**Figure 1:**
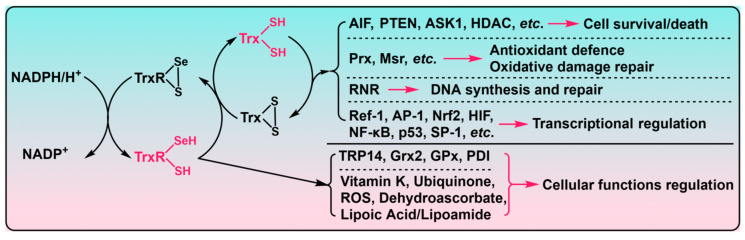
The principal functions of Trx system.

Trx is a small peptide protein, with a molecular weight of ~12 kDa, and it was initially discovered as the hydrogen donor for *Escherichia coli*’s ribonucleotide reductase (RNR) [11,12]. The human genome encodes for three specific Trx proteins, namely Trx1 in the cytoplasm and nucleus, Trx2 in mitochondria, and Trx3 or SpTrx (spermatozoa Trx) in testis [13,14,15]. All Trx proteins share a commonly canonical active site sequence as –Cys–Gly–Pro–Cys–(–CGPC–), which is located on a conserved typical structure named Trx fold [16,17]. The most studied mammalian Trx1 possesses its catalytic active site as –C^32^GPC^35^–, which functions as a redox switch for dithiol/disulfide exchange reactions. The human Trx1, yet, has three additional structural cysteine residues, i.e., Cys62, Cys69, and Cys73, which are all subjected to post-translational modifications contributing to the regulation of Trx1 functions [18,19]. Besides the only pair of active site cysteines, the mitochondrial Trx2 has an N-terminal extension import sequence that directs the protein to the mitochondria [20]. The major biological functions of the Trx system are carried out by Trx proteins through dithiol/disulfide exchange reactions, with a wide spectrum of downstream substrates (Figure 1). As an essential protein disulfide reductase, Trx proteins catalyze the reduction with rates being orders of magnitude faster than dithiothreitol or glutathione (GSH) [4]. The reduced Trx1 has the active site dithiol, and its N-terminal Cys32 with a low p*K*a value is deprotonated as a thiolate under physiological conditions [21]. The resulting thiolate could readily initiate the dithiol/disulfide exchange reactions with the oxidized substrate proteins, eventually resulting in the oxidized Trx1 and reduced substrate proteins. Finally, the TrxR1 catalyzes the transfer of two reducing equivalents from NADPH to the active site disulfide of oxidized Trx1, thereby completing the regeneration of the reduced Trx1 (Figure 1). 

Corresponding to the isoforms of mammalian Trx proteins, three isoforms of TrxR were identified in humans as well, that is, cytosolic and nuclear TrxR1 (encoded by *TXNRD1*), mitochondrial TrxR2 (encoded by *TXNRD2*), and testis-specific TrxR3 (also known as *t*hioredoxin *g*lutathione *r*eductase (TGR), owing to its ability to reduce glutathione disulfide and active site disulfide in oxidized Trx3, encoded by *TXNRD3*), all of which are selenoproteins that contain the distinctive 21st amino acid, selenocysteine (Sec, U) [14,18,21,22]. The human genome encodes 25 selenoproteins, generally having a single Sec residue in their catalytic active sites, the reasons for which are probably ascribed to the distinct chemical features of the selenium (Se) in Sec. Compared with its prevalent sulfur (S)-substituting congener, Cys, Sec is featured with several superior properties including a lower redox potential (−388 mV vs. −220 mV in Cys), lower p*K*a value (~5.2 vs. ~8.3 in Cys), and larger atomic radius (120 pm vs. 105 pm in Cys). These differences render Sec exceptionally reactive, as well as more resistant to overoxidation under physiological conditions [3,17], and this may also account for the higher catalytic activities of selenoenzymes in comparison with their corresponding mutated counterparts with Sec substituted by Cys. For example, U498C TrxR1 is 100-fold less catalytically reactive than its wild type, highlighting the indispensability of Sec for selenoenzymes in performing their biological functions [21]. 

Due to the extensive splicing occurring in *TXNRD* transcripts, many variants of TrxR proteins have been identified in different N-terminal domains [23]. The cytoplasmic splice variant TXNRD1_v1, commonly referred to as the “classical” form of TrxR1, represents the principal focus of investigations, being the most extensively studied isoform within the TrxR1 family. The second is the nuclear TXNRD1_v2, that is channeled to the nucleus for the regulation of nuclear transcription factors. The third variant of TrxR1 is TXNRD1_v3, which is anchored to cell membrane, leading to colocalization with membrane rafts and the formation of filopodia. The other two variants, TXNRD1_v4 and TXNRD1_v5, are less studied, and therefore, their functions are yet unrevealed. Similar to splicing in *TXNRD1* transcripts, *TXNRD2* and *TXNRD3* have also been confirmed in the existence of considerable splicing variants. 

**Figure 2:**
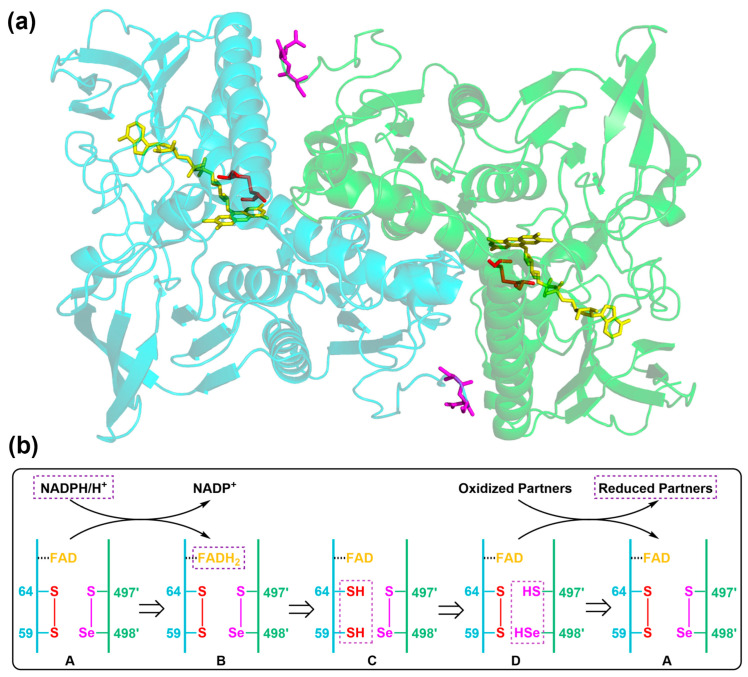
(**a**) The dimeric structure of human TrxR1 (PDB accession number: 3QFA, with two mutant sites, i.e., Cys497-to-Ser497 and Sec498-to-Cys498) [24]. One subunit is shown in cyan and the other is in green. The two subunits are arranged in a head-to-tail style. Molecule in yellow that binds to each monomer is FAD receiving electrons from NADPH. Residues in red are Cys59 and Cys64 in N-terminal active site –CVNVGC–, whereas residues in magenta are Ser497 and Cys498 in C-terminal active site –GSCG. (**b**) Cartoon representation of electrons flow in TrxR, which is indicated by the dashed boxes. The designations A-D correspond to different states of TrxR during the process of electron transfer. Arrows indicate the transformation of biological molecules from one status to other status.

TrxR belongs to the flavoenzyme class of the pyridine nucleotide disulfide oxidoreductase family, and it is structurally built up from a glutathione reductase (GR) skeleton as a homodimer arranged in a head-to-tail style (Figure 2a) [4,18]. Similar to GR, each subunit of mammalian TrxR has a FAD- and NADPH-binding domain in its N-terminus where the first evolutionarily conserved active site –CVNVGC– is located. Of note, besides a C-terminal interface domain, there exists an extension sequence of 16 amino acids in TrxR’s C-terminus which is absent from GR. Importantly, the second conserved active site –Gly–Cys–Sec–Gly–COOH (–GCUG) is positioned at this unique C-terminal extension with catalytic Sec residue being at the penultimate location. This flexible C-terminal catalytic active site, together with the “super” chemically nucleophilic Sec residue, dictates TrxR as an exceptionally accessible and reactive enzyme in biological contexts [17]. As a result, TrxR has a broad substrate specificity other than the native substrate Trx, such as a few additional Trx fold proteins like mitochondrial glutaredoxin (Grx2), glutathione peroxidase (GPx), protein disulfide isomerase (PDI), and several small molecules like selenite, selenocystine, lipoic acid, dehydroascorbic acid, and H_2_O_2_ (Figure 1) [21]. The TrxR-mediated catalytic transfer of electrons from NADPH to these substrates necessitates a head-to-tail arranged homodimer of TrxR [4]. The first active site disulfide in –CVNVGC– receives two electrons from NADPH via the enzyme-bound FAD in the same subunit, and then, the newly generated active site dithiol in the N-terminal –CVNVGC– reduces the C-terminal selenenylsulfide motif in the other subunit of the dimer TrxR, eventually resulting in a C-terminal reduced selenolthiol motif in TrxR that is easily accessible for its multiple substrates (Figure 2b). 

The Trx system is intimately implicated in propelling a multitude of reductive enzyme pathways and exerting redox modulation in a growing body of cellular functions, including cell survival, proliferation, differentiation, death, and many other physiological events [2,5,22,25,26,27,28,29,30]. TrxR utilizes NADPH to drive the biological functions of its downstream target proteins, mainly through Trx (Figure 1). Obviously, it seems safe to assume that TrxR orchestrates a cardinal role in the Trx system [3]. Indeed, a substantial number of studies revealed that an aberrant regulation of TrxR would lead to many pathological conditions [1,23,31,32,33,34,35,36,37,38]. As a consequence, it is of paramount importance to detect TrxR activity, for the purpose of further exploring its cellular functions in physiological processes and disentangling the unknown associations with potential pathological factors. 

**Figure 3:**
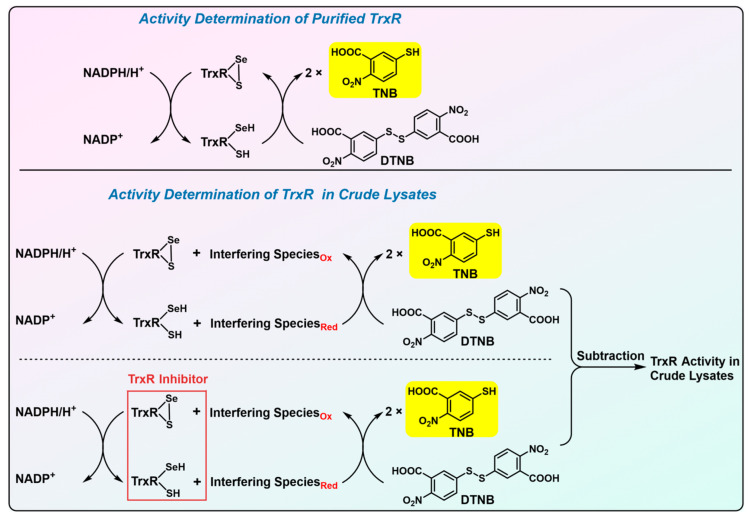
The principle of DTNB reduction assay and DTNB reduction assay in combination with TrxR inhibitors. Arrows indicate the transformation of biological molecules or reagents from one status to other status.

**Figure 4:**
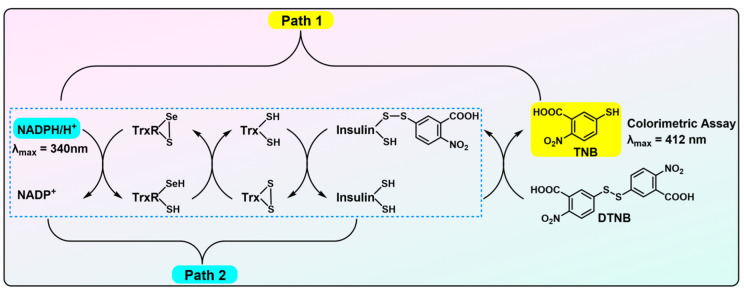
The principle of Trx-mediated insulin reduction assay. Path 1, also called endpoint insulin reduction assay, uses DTNB to quantify the newly generated sulfhydryl groups in insulin, and determination of TNB absorbance at λ = 412 nm indicates TrxR activity. Path 2, the dashed box in blue, shows an alternative approach to determinate TrxR activity, only by monitoring the NADPH decay at λ = 340 nm.

**Figure 5:**
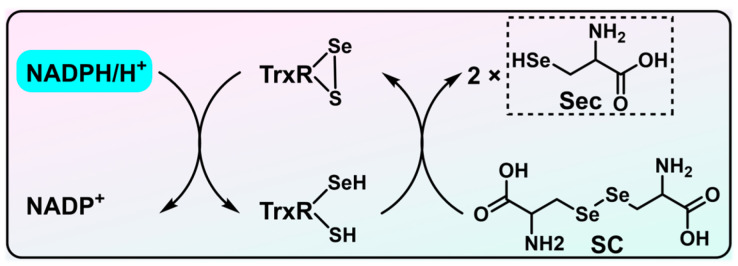
The principle of SC-TR assay. Selenocystine acts as the substrate of TrxR, and monitoring the decay of NADPH indicates the TrxR activity.

In the past few decades, various reliable assays for mammalian TrxR have been established and are commonly used in numerous laboratories [39,40]. Apart from its native substrate Trx, TrxR is capable of reducing a diverse spectrum of substrates. This fundamental attribute serves as the cornerstone for the development of these traditional assay methodologies. These assays are delineated in Figure 3, Figure 4 and Figure 5, and their underlying principles are briefly described in the subsequent discussions. 

Owing to the wide spectrum of TrxR substrates, including some small molecules [21], it is not surprising that 5,5′-dithiobis (2-nitrobenzoic acid) (DTNB, also known as Ellman’s reagent) could be directly reduced by TrxR at the expense of NADPH [41]. As shown in Figure 3 (upper panel), after the incubation of TrxR/NADPH with DTNB, a spectrophotometric determination of TNB (a bright yellow compound, characterized by the absorbance at λ = 412 nm) at pH = 8 is conducted, which reflects the TrxR activity. Obviously, this straightforward method is convenient for the activity determination of purified TrxR, but is not applicable in crude lysates inasmuch as substantial biological thiols in crude samples are also capable of reducing DTNB to TNB [39,40]. In order to tackle this issue, TrxR inhibitors, auranofin or aurothioglucose, were introduced in this assay [42]. As delineated in Figure 3 (bottom panel), performing the DTNB reduction assay with or without TrxR inhibitors can, thus, subtract background absorbance when determining the TrxR activity in crude samples. However, the reliability of the modified assay is stringently dependent on the selectivity of the TrxR inhibitors. Since the gold compounds are well established to covalently bind to other disulfide oxidoreductases and biological thiols [43,44,45,46], it is suggested to use more selective TrxR inhibitors to improve the precision of this method. Moreover, the two methods are not suitable for the activity measurement of low molecular weight (LMW) TrxRs in prokaryotes and lower eukaryotes (such as yeasts and plants), because the LMW TrxRs adopt a distinct catalytic mechanism from mammalian TrxRs [3,40]. 

The Trx-mediated insulin reduction assay was first developed by the Holmgren group and is considered the most reliable assay for TrxR activity [39,40,41,47,48,49]. As shown in Figure 4, Trx uses the reducing equivalents transferred from NADPH by TrxR to reduce the disulfide bonds in insulin. The newly generated thiols in insulin may be further titrated by DTNB to produce TNB in weak alkaline solutions. As it requires to terminate the reaction by DTNB in a solution containing a high concentration of chaotropic agents, this method is also frequently called endpoint insulin reduction assay (Path 1 in Figure 4). Alternatively, one can also measure the TrxR activity by monitoring the NADPH consumption at λ = 340 nm without terminating the reaction by adding DTNB (Path 2 in Figure 4). 

Noticeably, it is important to exercise caution because the resultant reduced insulin precipitates may confuse the absorbance readout at λ = 340 nm. Both assays (Path 1 and Path 2) are applicable to determine the TrxR activity in biological samples, by comparing the absorbance of TNB or decay of NADPH from the samples with and without an additional supplement of Trx. The assay shown in Path 1 is compatible with crude lysates, whereas the other in Path 2 may not be. Further, an exquisitely sensitive TrxR assay has been developed [50,51], predicated upon the observations that fluorescein isothiocyanate-labeled insulin (FITC-insulin) evinces a markedly greater affinity for Trx, as compared with unmodified insulin and manifests a conspicuous augmentation in fluorescence emission subsequent to the reduction of the disulfide bonds in FITC-insulin. In contrast to the classic insulin reduction assay, the modified methodology based on FITC-insulin demonstrates heightened sensitivity, allowing for the quantification of TrxR activity in biological specimens at the subpicomolar scale. 

As shown in Figure 5, TrxR utilizes NADPH to reduce *s*eleno*c*ystine (SC) to Sec, and one can monitor the consumption of NADPH at λ = 340 nm to reflect the TrxR activity [52]. The assay is featured with the convenience of being performed in either cuvettes or a microplate, measured by a traditional spectrophotometer or a plate reader, respectively, allowing one to readily carry out a high throughput screening using crude whole cell lysates. However, due to the abundance of other NADPH-dependent enzymes in cells, this method entails further improvement. Moreover, the millimolar concentration of GSH in cells may reduce the diselenide bond in SC [53,54,55,56,57,58], and this further adds to the inaccuracy of the assay. Several additional TrxR substrates, including juglone [59,60,61] and phenanthrene quinone [62], have also been utilized to establish assays for determining TrxR activity by monitoring the decay of NADPH at a wavelength of 340 nm. However, when implemented with crude lysates, these assays may suffer from non-specificity due to the potential use of juglone and phenanthrene quinone as substrates by NAD(P)H quinone oxidoreductase 1 (NQO1), which is also an NADPH-dependent enzyme. 

Moreover, various biochemical techniques exist for determining TrxR activity, including Trx-related protein of 14 kDa (TRP14)-linked cystine reduction [63], redox state-based SDS-PAGE/western blot, RT-PCR, ELISA, and matrix-assisted laser desorption/ionization imaging mass spectrometry (MALDI-IMS) [39,40]. However, these methods are subject to some limitations, such as elevated background noise, inadequate specificity, time and labor intensity, inconvenience, and high costs, to varying degrees. 

Most importantly, the methodologies outlined above for the detection of TrxR activity all require cell or tissue lysates, thus precluding in situ and real-time monitoring and imaging of TrxR within live cells or tissues. Furthermore, such approaches are not capable of detecting the effects of dynamic fluctuation in cellular redox balance on the oxidation–reduction status of TrxR, given that this selenoenzyme is rendered inactive upon oxidation. In contrast, fluorescent imaging based on small-molecule fluorescent probes may proffer solutions to the aforementioned issues involved in traditional assays. Organic small-molecule fluorescent probes exhibit a distinctive responsiveness to specific analytes, leading to a discernible alteration in their native fluorescence emission profiles. Endowed with intrinsic advantages, including high selectivity, remarkable spatiotemporal resolution, heightened sensitivity, noninvasiveness, and operational simplicity, organic small-molecule fluorescent probes designed for the specific in situ detection of diverse biological targets within viable cells have garnered escalating and pervasive interest [64,65,66,67,68,69,70,71,72]. Moreover, the spectroscopic properties of fluorescent probes could be readily tuned through the modification of their chemical structures. This additionally provides the huge potential for developing bespoke fluorescent probes possessing good selectivity and biocompatibility, a high signal-to-background fluorescence ratio, and prominent photoluminescence properties, e.g., a high quantum yield. 

In 2021, an excellent review article by Strongin et al. extensively commented on the TrxR fluorescent probes [73], primarily focusing on their synthesis, basic properties, and biological applications. However, considering the rapid development in this field, it is imperative to provide an updated analysis of the literature. In our study, we place a particular emphasis on thoroughly analyzing the reaction mechanisms, construction strategies, and potential limitations associated with the existing TrxR probes. Furthermore, we emphasize the critical challenges involved in future TrxR fluorescent probe development and present valuable insights and perspectives to guide their further advancements.

## 2. The Mammalian TrxR Probes 

During the recent decades, there has been a significant surge of interest in the advancement of small-molecule fluorescent probes for imaging and detecting numerous biological targets [64,67,69,74,75,76]. While a small subset of the probes may exhibit limitations in terms of target specificity, the majority of these pioneering probes have increasingly emerged as remarkably powerful tools for the real-time monitoring, in situ visualization, and quantitative analysis of various dynamic biological processes in live cells or tissues. This is attributed to their high specificity, exceptional sensitivity, non-destructive nature, and rapid analytical characteristics. Moreover, the modifiable chemical structures of small -molecule fluorescent probes enable the real-time visualization and detection of diverse ranges of enzymatic locations and activities in their native environments. The past few years have witnessed the rapid development of TrxR probes, and the arsenal of TrxR probes has become gradually enriched. These probes have contributed significantly to the investigation of TrxR’s biological functions and have been valuable tools in TrxR-related research. To furnish readers with a quick and concise overview of these probes, we categorize them into distinct classes according to their different recognition parts/triggers. 

### 2.1. Strained 1,2-Dithiolane as the Trigger 

**Figure 6:**
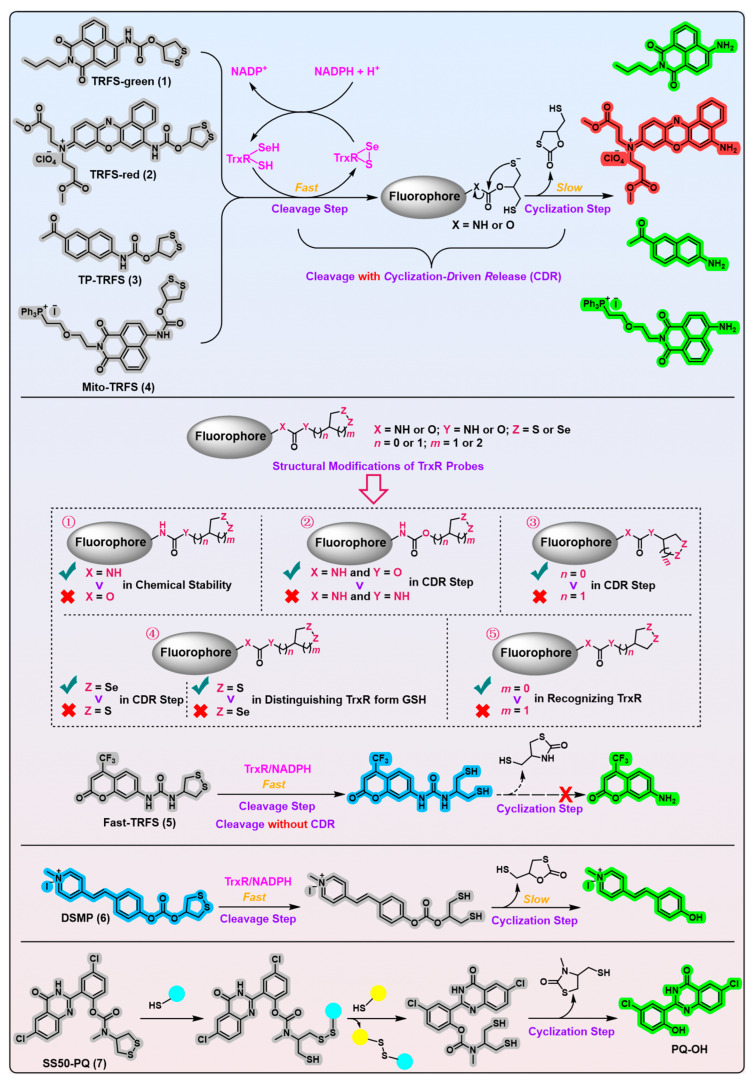
1,2-Dithiolane-based TrxR probes. Probes 1–4 employ carbamate motif as the linker. Probes 5 and 6 use urea and carbonate motif as the linkers, respectively. The model probe 7 uses a stable phenolic carbamate motif as the linker. Following a two-step process, the free fluorophores are liberated in probes 1–4 and 6–7. Probe 5 is triggered only by cleavage of the disulfide bond in the recognition part. ①–⑤ indicate the influences of changing atoms in the linker unit and recognition part on the properties of probe 5. ①–⑤ also show the development process of probe 5. The cyan and yellow balls having sulfydryl on them shown in the working process of probe 7 indicate various biological thiols. Protein TrxR is shown in pink. The colors covering the chemical structures show their fluorescence colors after TrxR activation, and the gray indicates the fluorescence of the probes has been quenched.

Enlightened by the early finding that the five-membered cyclic disulfide compounds lipoamide and lipoic acid could be efficiently opened by mammalian TrxR but not GSH [77], the Fang group developed the first off–on fluorescent probe, called TRFS-green (Figure 6, probe 1) [78], for mammalian TrxR by linking the fluorophore naphthalimide to an artificial cyclic disulfide 1,2-dithiolane via a carbamate linker. With the reduction by NADPH under the catalysis of TrxR, TRFS-green emits a bright green fluorescence signal at λ = 538 nm when it is excited at λ = 438 nm. TRFS-green shows favorable selectivity to TrxR over other biologically related species, including small molecules like NADPH, GSH, ascorbic acid, and enzymes like lipoamide dehydrogenase and GR. Importantly, TRFS-green shows a trivial fluorescence signal upon U498C TrxR treatment, underlining the critical significance of TrxR’s Sec residue in recognizing TRFS-green. Further, lysates of different cell types with varying TrxR activity (determined by the classic Trx-mediated insulin reduction assay) show a corresponding ability to proportionally switch on the fluorescence signal of TRFS-green, whereas the TrxR inhibitor 2,4-dinitrochlorobenzene (DNCB) dose-dependently darkens the TRFS-green, highlighting the involvement of cellular TrxR in turning on the TRFS-green. 

However, the TRFS-green suffers the drawbacks of requiring a lengthy time (~4 h) to plateau, coupled with a modest increase (~25-fold) in emission intensity upon treatment with TrxR. This arises from the sluggish cyclization step, as elucidated by a mechanistic study of TRFS-green employing LC-MS analysis [53]. In addition, the development and validation processes of TRFS-green encounter several significant concerns that warrant attention: (1) The activation of TRFS-green necessitates the use of a non-physiological concentration of TrxR (200 nM) to achieve a substantial ~25-fold fluorescence increment. Even when treated with 50 nM of TrxR, which still surpasses the physiological concentration of TrxR (~20 nM), only a ~5-fold fluorescence increment is observed after an extended 3 h treatment. In addition, 1 mM of GSH, which is far below the physiological concentration (~5 mM), was used to evaluate its influence on TRFS-green. This deviation from physiological conditions raises doubts regarding the reliability of TRFS-green’s selective response to TrxR. (2) The interference test conducted on TRFS-green fails to explore its response to several crucial redox enzymes such as Trx, GR, Grx, and TRP14. Additionally, the verification of TRFS-green’s specificity lacks the adoption of fully integrated redox cycling enzymatic systems, such as GSH, Trx, and Grx systems. These omissions, as explicitly emphasized by Thorn-Seshold et al. [79,80], may deteriorate the reliability of TRFS-green’s selectivity claims. Ignoring interferences from these vital redox players seriously undermines the confidence in TRFS-green’s ability to accurately detect TrxR. (3) DNCB, as an inhibitor for TrxR, exhibits inadequate selectivity due to its promiscuous reactivity with various biological thiols. It is imperative to utilize a more specific TrxR inhibitor, such as TRi-1 [81], to validate the selectivity of TRFS-green [79,80,82]. While TRFS-green has been prevalently applied in many studies to probe the cellular status of TrxR [83,84,85,86,87,88,89,90,91,92,93,94,95], caution and reevaluation are warranted in interpreting these findings. This is due to the seminal work by Thorn-Seshold and colleagues, which unequivocally demonstrates the significant contributions of Trxs, Grxs, and TRP14 to the signal generation observed from TRFS-green, in addition to TrxR [80]. Interestingly, a recent study by Lu et al. reported TRFS-green is also a substrate of bacteria Trxs and Grxs; however, only the complete or intact thiol-dependent redox systems could activate the probe [96]. In other words, the individual bacteria TrxR, GR, and GSH do not trigger TRFS-green. Notably, *E. coli* Grx2 and Grx3 were found to have higher activity than other redoxins to light up TRFS-green in Trx- or Grx-null mutant strains. The TrxRs of prokaryotes are LMW proteins that do not have Sec in their active sites, whereas the TrxRs of higher eukaryotes are the opposite [3,4,17]. This structural distinction potentially elucidates the inherent ability of mammalian TrxR to activate TRFS-green. Notably, a significant degree of evolutionarily conserved similarity exists among Trxs spanning from bacteria to mammals. Hence, it is not surprising that mammalian Trxs, akin to their bacterial counterparts, can effectively activate TRFS-green, as convincingly demonstrated by the Thorn-Seshold group [80]. In addition, Thorn-Seshold et al. also revealed the robust activation of TRFS-green by mammalian Grxs [80], a finding that aligns to some extent with the outcomes of Lu’s investigation. Thus, the selectivity of TRFS-green necessitates reconsideration and enhancement. Collectively, these findings suggest TRFS-green is an unreliable indicator of TrxR activity.

Fluorescent probes with an emission wavelength greater than 600 nm are featured with an enhanced tissue penetration depth and higher spatial resolution, due to a diminished background autofluorescence from tissue [97,98,99,100]. On this account, a further improvement of TRFS-green by the same group leads to the generation of TRFS-red (Figure 6, probe 2) [101], a red emission off–on fluorescent probe (λ_em_/λ_ex_ = 660/615 nm) that maintains favorable selectivity to mammalian TrxR. TRFS-red is structurally constructed from the TRFS-green scaffold but has a Nile blue core as the fluorophore. Surprisingly, upon treatment with TrxR, TRFS-red has a higher response rate (~2 h) to plateau and a larger fluorescence increment (~90-fold) compared with the parent probe TRFS-green. The reasons for the improved performance of TRFS-red may be attributed to two aspects: (1) the Nile blue is a cation molecule, which facilitates the electrostatic interaction of TRFS-red with the negatively charged C-terminal active site of TrxR; (2) The strong electron-withdrawing iminium moiety in the Nile blue endows it with a good leaving ability, which hastens the cyclization step during the working process of TRFS-red. Indeed, TRFS-red releases a strong red fluorescence signal after incubation with HeLa cells for 1 h, whereas TRFS-green gives a detectable fluorescence signal after a 4 h incubation in cells. HPLC analysis shows TRFS-red works through the same mechanism as TRFS-green. Another work for the improvement of TRFS-green was the development of the first off–on two-photon fluorescent probe for mammalian TrxR, TP-TRFS (Figure 6, probe 3; λ_em_/λ_ex_ = 490/370 nm) [102]. Linking the recognition part 1,2-dithiolane to a two-photon fluorophore 2-acetyl-6-aminonaphthalene furnishes TP-TRFS. The probe retains discernible selectivity towards TrxR, when compared with other cellular entities, including Trx, GR, GSH, NADPH, esterase, and others. Notably, upon a 3 h treatment with TrxR leading to a plateau, the probe exhibits a remarkable 15-fold increase in fluorescence intensity. Since two-photon fluorescence imaging, compared with conventional one-photon, is featured with deeper tissue penetration, higher spatial resolution, and less damage to tissues, two-photon fluorescent probes are suitable for a long-duration study of TrxR in tissues and live organisms [103,104,105]. Indeed, TP-TRFS was successfully applied in imaging TrxR in live zebrafish. Meanwhile, the probe helps to disclose the distinct loss of TrxR activity in the brain of a mouse model of stroke, indicating the intimate implication of TrxR malfunction in the pathophysiology of stroke. Notwithstanding the aforementioned merits of TRFS-red and TP-TRFS, they face analogous challenges encountered by TRFS-green throughout their development and validation processes, thereby substantially compromising their ability to selectively detect TrxR in cells and live organisms. 

Mitochondrial TrxR (TrxR2) is crucial in maintaining redox homeostasis and regulating many vital signaling pathways in mitochondria [13,106]. As a major source of cellular ROS, dysfunction of mitochondria probably gives rise to oxidative stress in cells, which is the causal factor for various diseases such as neurodegenerative diseases, cancers, and cardiovascular diseases [106,107,108,109]. Therefore, precisely detecting the status of TrxR2 is important for both exploring its physiological role in normal conditions and interrogating its potential relation with many diseases in pathological conditions. For this purpose, Fang et al. developed a mitochondria-targeted TrxR2 probe, Mito-TRFS (Figure 6, probe 4; λ_em_/λ_ex_ = 540/438 nm) [110]. It is structurally similar to TRFS-green but contains an additional mitochondria-targeting triphenylphosphonium moiety. Mito-TRFS likewise exhibits a certain specificity for TrxR over other functionally related biological species, including GR, U498C, GSH, and others. Moreover, the fluorescence colocalization assay provides evidence of its selectivity for imaging TrxR2 within live cells. Compared with the mother probe TRFS-green, Mito-TRFS is characterized by a faster response rate (~1 h) and higher fluorescence increment (~40-fold) towards TrxR. This presumably results from the relatively high hydrophilicity and positively charged triphenylphosphonium of Mito-TRFS, which subserves its binding with the negatively charged C-terminus of TrxR2 via an electrostatic interaction in aqueous mitochondrial matrix. The biological utilization of Mito-TRFS unveils a substantial impairment of TrxR2 activity within a cellular model of Parkinson’s disease. However, Mito-TRFS not only shares similar adverse issues encountered by TRFS-green but also exhibits certain design-related shortcomings. With the incorporation of a triphenylphosphonium moiety into the fluorophore, Mito-TRFS retains its intrinsic mitochondrial targeting capability even after potential activation or liberation in the cytosol by TrxR1 or other species. This raises further uncertainties regarding the ability of Mito-TRFS to effectively target TrxR2. 

In 2019, the Fang group conducted a comprehensive mechanistic study of the TRFS-green activation process and found that the reason accounting for the slow response of TRFS-green is its sluggish cyclization step [53]. The rationale seems to emerge that the rational modulation of the structural determinants that influence the response rate and specificity of the probes towards mammalian TrxR may generate TrxR probes with improved properties (Figure 6, probe 5). Then the authors performed a systematic structural modification of TRFS-green to fully clarify the structural factors [53]. As shown in Figure 6, the evaluation of the probes, which were generated via tuning the linker units and recognition parts, contributes to several interesting findings: (1) when the fluorophore is –OH-containing (X = O), both the carbonate- and carbamate-based probes are chemically unstable due to E1cB elimination [80]; (2) a carbamate linking unit (X = N and Y = O) is more preferred than a urea linking unit (X = N and Y = N) in promoting the cyclization step; (3) the cyclization step resulting in the formation of a six-membered thiocarbonate (*n* = 1) is drastically hampered compared with TRFS-green (*n* = 0), more easily liberating a five-membered thiocarbonate; (4) diselenide (Z = Se) in a recognition moiety is more favored than disulfide (Z = S) in accelerating the cyclization step, whereas diselenide is less specific in distinguishing TrxR form GSH in comparison with disulfide; (5) a six-membered disulfide (*m* = 1) recognition moiety resists reduction by TrxR, compared with a five-membered one (*m* = 0), thereby showing less selectivity for TrxR over other biological species; and (6) disulfide/diselenide bonds can quench the emission of certain fluorophores, and thus, a direct cleavage of the bonds turns on the fluorescent signal of the probes. The last unexpected finding leads to the establishment of a novel sensing mechanism that confers a superfast response to TrxR (or another stimulus of interest), absent from undergoing the sluggish cyclization step. Following the design strategy of TRFS-green, the authors identified the Fast-TRFS probe (λ_em_/λ_ex_ = 460/345 nm), which shows favorable selectivity for TrxR, concurrent with an exceptionally fast response rate. Due to the stability of urea linkage, switching on the Fast-TRFS is achieved by simply cleaving the disulfide bond without the process of further cyclization. Theoretical calculations suggest that the non-fluorescent behavior of Fast-TRFS can be attributed to the *p*hoto-induced *e*lectron *t*ransfer (PET) process, initiated by the presence of a disulfide bond within the recognition moiety. Conversely, the cleavage of the disulfide bond extinguishes the PET process, leading to the liberation of the probe’s fluorescence. Similar to other TRFS series probes, while Fast-TRFS demonstrates negligible susceptibility to many biological species including Trx, GR, GSH, NADPH, Sec, diaphorase, amide hydrolase, and others, the probe remains devoid of comprehensive cross-validation and the implementation of a fully redox cycling enzymatic system to assess the interferences from corresponding species. Notably, Fast-TRFS exhibits a 55.7-fold selectivity for TrxR (50 nM) over cellular abundant GSH (1 mM), in a stark contrast to TRFS-green (15.6-fold) and TRFS-red (12.8-fold). Compared with other TRFS series probes, a faster answer rate (~5 min) and higher fluorescence increment (~80-fold) in response to TrxR are distinct characteristics of Fast-TRFS, whereas TRFS-green and TRFS-red show a less than 20-fold increase within 15 min. 

Interestingly, Lu et al. recently showed that Fast-TRFS is also a rapid reporter for the bacteria Trx and GSH system, but not for TrxR, GR, and GSH [111]. It means that Fast-TRFS is a substrate for bacteria Trxs and Grxs. In comparison, the study showed the bacterial Trx system has a higher reaction rate and is responsible for the fast disulfide exchange reaction, whereas the GSH system belongs to a slow response network in bacteria. The study also indicates Fast-TRFS as a fast reporter for the bacterial Trx system. Given the considerable evolutionary conservation observed among Trxs across different species, ranging from bacteria to mammals, mammalian Trxs are believed to hold a great potential to activate Fast-TRFS within a fully cycling enzymatic system. Further, owing to the distinct structural attributes and working mechanisms of bacterial and mammalian TrxR, Fast-TRFS is expected to manifest a superior response to mammalian TrxR. This is attributed to the presence of a flexible, exposed C-terminal active site containing Sec on the surface of mammalian TrxR, which represents a unique characteristic absent in bacterial TrxR [3,4,14,17]. Additionally, bacterial TrxR requires a conformational rotation to expose its buried active site and then exclusively catalyzes the reduction of its own Trx. Collectively, these factors suggest that mammalian TrxR may exhibit more favorable kinetics towards Fast-TRFS, compared with bacterial TrxR. However, it is important to note that the selectivity of Fast-TRFS towards mammalian TrxR still entails further scrutiny, reassessment, and refinement. 

Most recently, Zhou et al. developed a styrylpyridinium-based two-photon ratiometric fluorescent probe for TrxR2 (Figure 6, DSMP, probe 6) [112], which possesses the advantages of a fast response (plateaus within 12 min), large Stokes shift (λ_em_/λ_ex_ = 510/340 nm), and ability to penetrate the blood−brain barrier. Compared with single emission imaging, ratiometric imaging through two different emissions is more preferred owing to its inherent calibration of interference to circumvent distorted data [113]. Different from carbamate-linked TRFS probes, probe DSMP is carbonate-bridged with 1,2-dithiolane as the recognition part. Importantly, this carbonate-bridged probe exhibits hydrolytic robustness in TE and PBS buffers at an ambient temperature for 3 h. DSMP shows a resistance to a variety of biological species, including GSH, Cys, H_2_S, ascorbic acid, NADPH, etc.; however, the concentrations of certain species are too low to reach the physiological levels, e.g., GSH at 100 μM indeed has a negligible effect on DSMP, whereas its physiological concentration (~1–10 mM) would activate DSMP (*information obtained from personal communication with the authors)*; this result is also consistent with the structure–activity relationship summarized from Fast-TRFS [53]). DSMP exhibits a favorable binding to TrxR, with *K*_m_ = 12.5 ± 0.2 μM, and a ~4-fold fluorescence increment is achieved upon TrxR treatment. The fluorescence colocalization assay reveals that DSMP primarily localizes within the mitochondria; however, the Pearson’s correlation coefficient (0.74~0.85) below 0.9 may detract from its high spatial resolution imaging in vivo. The biological application of DSMP enables one to distinguish normal cells from cancer cells, due to the overexpressed TrxR2 in cancers, and it also uncovers the drastic function loss of TrxR2 in a cellular Parkinson’s disease model. Due to the two-photon property of DSMP, the probe largely facilitates the functional detection of TrxR2 in brain tissues in a mouse model of ischemic stroke induced by a middle cerebral artery occlusion. Despite the many advantages of the DSMP probe, it still shares similar challenges as observed in the TRFS series probes. These challenges primarily pertain to the lack of in-depth cross-validation utilizing fully integrated redox enzymatic cycling systems, specific TrxR inhibitors, additional redox enzymes, and other assays necessary to thoroughly assess the probe’s genuine selectivity towards TrxR2. Moreover, similar to Mito-TRFS, DSMP also exhibits the potential to undergo activation and a subsequent fluorescence release by TrxR1 within the cytosol prior to its translocation to the mitochondria. Consequently, these factors raise substantial concerns regarding the capability of the DSMP probe to accurately discern TrxR2 in cells or tissues. 

A recent study from the Thorn-Seshold group expressed concern about the specificity of 1,2-dithiolane-based TRFS series probes [80]. The authors designed and synthesized a 1,2-dithiolane-based model probe SS50-PQ (Figure 6, probe 7) to explore whether 1,2-dithiolane is selective for TrxR, with TRFS-green (Figure 6, probe 1) and Fast-TRFS (Figure 6, probe 5) as controls. Triggering the SS50-PQ is exclusively through the cyclization-driven release of the fluorophore PQ-OH, due to the photoluminescence mechanism of an *e*xcited-*s*tate *i*ntramolecular *p*roton *t*ransfer (ESIPT). Hence, the turn-on process of SS50-PQ is independent of any environment effects. Together with its large Stokes shift property, SS50-PQ is indeed a sensitive and interpretable probe for 1,2-dithiolane cleavage. On the one hand, the authors hypothesized that the recognition moiety of a probe serves as the key determinant governing its reactivity and selectivity. Consequently, the results obtained from the employment of the 1,2-dithiolane-based model probe SS50-PQ are deemed suitable and valid for interpreting any 1,2-dithiolane probes. Remarkably, their hypothesis aligns well with the subsequent cell-free experimental findings, which demonstrate that SS50-PQ exhibits tangible responses towards an extensive range of biological species, including TrxR, TRP14, Grx, GSH, Cys, N-acetylcysteine (NAC), cysteamine, and several others. Additionally, TRFS-green is also activated by TrxR, Trx, Grx, and TRP14, further corroborating their hypothesis. On the other hand, considering the perspective of chemical biology, it is important to note that the interaction between the probe and the target protein extends beyond the mere recognition part of the probe and the active site of the protein. In fact, the involvement of both the fluorophore and linker unit in their interaction with the surrounding residues of the target protein holds significant relevance [114,115,116]. In addition, as Fang et al. revealed, the properties of trigger-cargo releasing systems are significantly influenced by their linker unit [53]. In this sense, the influences of a reducible trigger, a linker unit, and a fluorophore are intricately intertwined and cannot be simply separated and dealt with in isolation. 

**Figure 7:**
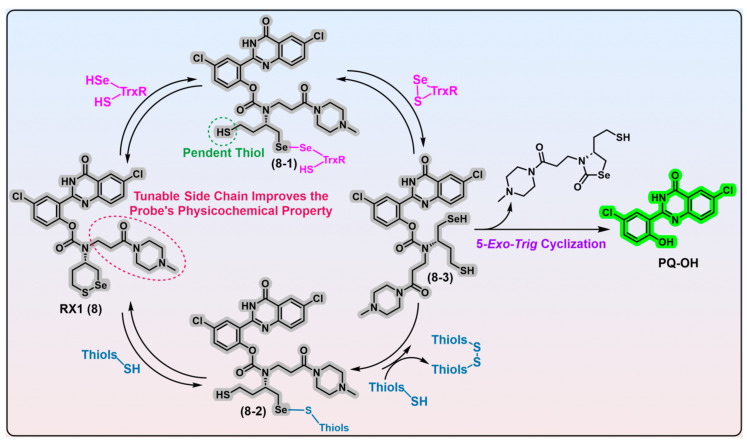
1,2-Thiaselenane-based TrxR1 probe RX1. Probe RX1 interacts with the desired TrxR1 to liberate the fluorescent PQ-OH, during which process the key intermediates 8-1 and 8-3 are generated. Probe RX1 can also react with high concentrations of monothiols to give intermediate 8-2; however, this intermediate encounters significant kinetic and thermodynamic barriers to yield key intermediate 8-3. Thus, this unwanted parallel pathway induced by monothiols does not trigger the RX1. The bidirectional arrows indicate corresponding transformations are reversible. Protein TrxR1 is shown in pink and various biological thiols are shown in blue. Dashed ellipse in red shows the tunable side chain of probe 8, and the corresponding words in red explain the function of the side chain. Dashed cycle in green shows the potential reactive thiol in intermediate 8-1, and the corresponding words in green show the name of thiol appearing in the main text. The colors covering the chemical structures show their fluorescence colors after TrxR activation, and the gray indicates the fluorescence of the probes has been quenched.

In the further cellular evaluations of SS50-PQ and TRFS-green, the authors used SS00-PQ (a TrxR-independent linear disulfide probe) and RX1 (Figure 7, probe 8; the most recently reported highly specific mammalian TrxR1 probe [79]) as the controls. The results come that SS00-PQ, SS50-PQ, and TRFS-green are indeed almost independent of cellular TrxR modulation via chemical upregulation/knockdown, genetic knockout, and specific TrxR inhibitor treatment; however, the RX1 probe is significantly affected by these modulations. Although TRFS-green shows mild sensitivity to the genetic modulation of TrxR, it is important to highlight that TRFS-green retained its activation by 60% even in cells with aa complete knockout of TrxR1. Given the minimal response in TRFS-green to various TrxR modulations, the sensitivity of TRFS-green to Au-based treatment may be ascribed to the lipophilicity of Au compounds, such as auranofin, which enables them to effectively block cellular membrane thiols. Consequently, the diminished fluorescence of TRFS-green after a treatment of Au compounds may be a result of inhibited cellular uptake rather than the direct inhibition of TrxR by Au compounds. In combination with the aforementioned cell-free results, it is plausible to draw the inference that all existing carbamate-bridged 1,2-dithiolane probes may not possess selective cellular reporting capabilities for TrxR, irrespective of the observed partial reliance of TRFS-green on TrxR as evidenced by genetic studies [78,80]. 

Further, the authors evaluated Fast-TRFS (Figure 6, probe 5) and speculated that the probe may undergo a reduction-independent but concentration-dependent and strain-promoted *r*ing-*o*pening *p*olymerization (ROP) to emit fluorescence, which is quite different from the previously reported activation mechanism [53]. The authors hypothesized that the hydrophobic Fast-TRFS would concentrate from the cellular aqueous phase to the apolar cellular lipid membrane of the high surface area, thus initiating ROP to form a polymer of Fast-TRFS. According to the activation mechanism of cleavage without a cyclization of Fast-TRFS (Figure 6, probe 5) [53], the polymer of Fast-TRFS would emit the fluorescence, and furthermore, this fluorescence intensity would be lipid concentration-dependent. The authors utilized small lipid/phospholipid vesicle suspensions to simulate the apolar environments of cellular membrane, and they then evaluated the performance of Fast-TRFS in this artificial medium. Indeed, the surface phenomena observed in this experiment are congruent with the proposed hypothesis. Nevertheless, there persist a number of essentially notable concerns regarding both the hypothesis and the experimental outcomes. First, Fast-TRFS is a superfast reporter of TrxR (~70-fold fluorescence intensity plateaus within 5 min in cellular conditions), whereas the ROP-mediated turn-on of Fast-TRFS plateaus after a 60 min treatment even in a high concentration of lecithin (10 mg/mL). Second, the liberated fluorescence of Fast-TRFS is evenly distributed in the cytosol [53], whereas the ROP-induced fluorogenicity of Fast-TRFS is believed to be localized to the cellular membrane. Third, although the use of this artificial, cell-like environment devoid of TrxR may be more tractable, it fails to accurately emulate the inhomogeneous cellular context, as TrxR probably possesses superior kinetics when compared with other mechanisms responsible for lighting up Fast-TRFS. Fourth, for TRFS-green type probes working via the mechanism of cleavage with cyclization, the ROP-induced fluorescence emission wavelength (λ_em_ = ~490 nm for TRFS-green type probes when cleaving without cyclization [53]) or any other intact probe- or membrane thiol exchange intermediates/adducts-induced environment-dependent fluorescence emission wavelength is different from the emission wavelength of free fluorophores (λ_em_ = 538 nm). This fact makes the hypothesis of ROP-mediated fluorogenicity non-universal, i.e., except for Fast-TRFS (Figure 6, probe 5) with a unique activation mechanism, other TRFS probes all show the free fluorophores’ emission wavelength in cells but not the ROP-induced fluorescence emission wavelength. 

While numerous fluorescent probes, including the majority of TrxR probes, may exhibit potentially interfering optical turn-on effects, such as the environment sensitivity of certain fluorophores, it remains obscure whether these effects competitively or comparably influence the fluorescence signal triggered by TrxR. On one hand, the existing 1,2-dithiolane probes have been found to lack sufficient specificity for TrxR thus far. On the other, it is noteworthy that 1,2-dithiolane probes incorporating different fluorophores and linkages display significantly distinct fluorogenic properties. These observations suggest that the reducible trigger of a trigger-cargo platform indeed executes a pivotal role in selectively recognizing the desired biological targets. Nevertheless, it is also important to acknowledge that the reducible trigger, the linker unit, and the fluorophore all have an impact on the ultimate fate of a probe. Therefore, substantial efforts are still required to delve deeper into the biochemical aspects of 1,2-dithiolane on multiple levels, with the anticipation that its new forthcoming derivatives may unexpectedly emerge to be validated as truly specific to TrxR. 

Importantly, this work also possesses noteworthy merits that deserve significant recognition and advocacy [80]. The modular reduction-triggered phenolic carbamate platform (Figure 6, probe 7) developed by the authors has the following advantages: (1) the Haugland’s precipitating fluorophore PQ-OH (Figure 6, probe 7) is featured with a large Stokes shift (λ_em_/λ_ex_ = 530/360 nm) that ensures the platform has a high spatial resolution [117,118]; (2) The PQ-OH-based platform switches on its ESIPT-based fluorescence signal only when cleavage with CDR takes place, indicating that the environment-dependent optical turn-on effects no longer interfere the signal generation. Furthermore, this phenomenon may contribute to the high signal-to-background fluorescence ratio observed; (3) The use of a phenol-based cargo release offers several attractive advantages, including a wider selection of potential cargos and more favorable leaving kinetics when compared with an aniline-based release. A key benefit of this approach is the ability to mask the hydroxyl group of many fluorophores, drugs, and agents, which can otherwise quench their fluorescence or activities. This allows for the construction of high-resolution probes, as well as the development of conceptually true prodrugs/theranostic agents. 

The diversification of recognition parts to create novel redox substrates that are specific to diverse oxidoreductases is a challenging yet highly promising research field. The incorporation of selective triggers for various oxidoreductases such as GPx, GR, Trx, Msr, Prx, etc., into this modular platform presents an exciting opportunity to expedite the identification of high-quality probes. This approach can greatly facilitate and accelerate the study of redox biology. Furthermore, expanding this modular platform would have additional benefits, including the construction of oxidoreductase-targeted prodrugs and theranostic agents.

### 2.2. 1,2-Thiaselenane as the Trigger

Previous studies revealed that both the topology (linear or cyclic) and geometry (strained or stabilized) of disulfide exert profound effects on the cellular performance of disulfide-based probes, as both characteristics significantly influence the sensitivity of the probes to background monothiols [119]. Theoretically, disulfide probes with linear topology exhibit kinetic irreversibility against cellular monothiols in high concentrations (~1–10 mM). Disulfide probes with cyclic topology appear to demonstrate varying degrees of resistance to background monothiols [53,120]. Theoretically, strained cyclic probes are susceptible to interference from monothiols due to the inherent kinetic lability of strained cyclic disulfides. Conversely, non-tensile cyclic probes typically remain stable against monothiols attack via reversible thiol–disulfide exchange reactions. Interestingly, alicyclic six-membered disulfides were recently reported as selective substrates of Trx by the Thorn-Seshold group [119,121]. In this sense, 1,2-dithianes may be tuned and harnessed for probe designs, acting as specific triggers for various oxidoreductases. 

Most recently, based on their recently developed modular reduction-triggered phenolic carbamate platform [80], the Thorn-Seshold group successfully developed an exceptionally selective mammalian TrxR1 fluorescent probe (Figure 7, RX1, probe 8) that employs a novel stereoisomeric 1,2-thiaselenane as the recognition moiety. Given the catalytic principles of TrxR and monothiols, the replacement of a sulfur atom in 1,2-dithiane with a selenium atom may be advantageous for facilitating the selective recognition of TrxR as well as strengthening resistance against monothiols. Experimental evidence suggests that it is Sec in TrxR1 and a selenium atom in the 1,2-thiaselenane of RX1 probe that are both kinetically and thermodynamically preferred in the first recognizing exchange reaction step, which gives the key intermediate 8-1 (Figure 7). Despite the presence of a pendent thiol, the intermediate 8-1 displays significant chemical resistance to undergo a 6-*exo*-*trig* “on-reductant” cyclization. Instead, it exhibits a strong preference for a kinetically smooth reaction, whereby the nascent diselenide bond of intermediate 8-1 is attacked by the thiolate in the active site of TrxR, ultimately resulting in the completion of the catalytic cycle of TrxR and the generation of the fully reduced intermediate 8-3. While the reductive intermediate 8-3 is capable of reducing the disulfide biomolecules accessible within cells, there appears no fluorescent signal in this process. Furthermore, due to the inherent nucleophilicity of its selenolate moiety, intermediate 8-3 can readily undergo a chemically favored 5-*exo*-*trig* cyclization, resulting in the release of the fluorescent PQ-OH molecule. Alternatively, although the reaction of RX1 with high levels of background monothiols can generate intermediate 8-2, the subsequent extrusion of the fully reduced intermediate 8-3, propelled by a second molecule of monothiol, is extremely disfavored in both kinetics and thermodynamics. Based on these observations, it can be inferred that RX1 exhibits strong kinetic and thermodynamic selectivity for TrxR, effectively precluding the interference from high levels of monothiols present within cells. In other words, the selenium present in the trigger of RX1 serves two distinct functions: (1) facilitating the recruitment of the desired TrxR in cells and (2) acting as a sink for the monothiols-induced parallel pathway, thereby preventing unwanted interference. Further LC-MS analysis provides additional confirmation of this underlying mechanistic process. In vitro evaluation of RX1 reveals its strong selectivity towards the TrxR1 isoform, as evidenced by the inability of the Sec-deficient U498C TrxR1 to trigger RX1. This observation highlights the essential role of selenium in TrxR1 for the recognition and cleavage of the selenenylsulfide bond present within RX1. The cellular modulation of TrxR1 via genetic knockout/knock-in, chemical inhibition, and selenium supplementation/starvation further underscores the high selectivity of RX1 towards cellular TrxR1. Importantly, high concentrations of monothiols such as GSH, as well as protein thiols such as GR, Grx, and TRP14, have been shown to have a minimal impact on the efficacy of RX1, further accentuating its exceptional selectivity for TrxR1. 

Notwithstanding these findings, RX1 may have some minor defects in selectivity, as Trx isoforms appear to be effective in switching on RX1 even below physiological concentrations. On one hand, experimental evidence indicates that while Trxs exhibit an approximately 50-fold slower activation of RX1 than TrxR1, the approximately 1000-fold higher concentration of Trxs relative to TrxRs in cells may compensate for this kinetic disadvantage. On the other hand, Trxs possess a significantly greater number of downstream partners in cells compared with TrxR1, potentially providing an opportunity for RX1 to selectively target TrxR1. In addition, whether RX1 is responsive to free Sec remains unknown. The clarification of the impact of Sec in activating RX1 holds significant importance, as the presence of the Se atom in the trigger of RX1 promotes its favorable recruitment of highly nucleophilic species. This recruitment also bears the potential risk of an undesired activation of RX1. These negative effects, working in combination with RX1′s slow response rate (~3 h to complete the turnover of 20 nM TrxR1), create a more complex and problematic situation in cells that may weaken the RX1’s selectivity for TrxR1. Regardless of these minor problems, RX1 is overall a highly selective probe for TrxR1. The N-derived side-chain of RX1 can be modified to adjust its solubility, which is advantageous for its practical use. Furthermore, the modular reduction-triggered PQ-OH-based carbamate platform offers several other benefits that facilitate the quantitative high-throughput screening of TrxR1 inhibitors in live cells using RX1 [80]. Due to the exceptional selectivity of RX1 for TrxR1 in cells, it is an efficient tool for identifying specific inhibitors of TrxR1 at the cellular level, by comparing the potency of a compound in inhibiting TrxR1 in both cell-free and live cell conditions. Promisingly, RX1 is poised to actuate drug discovery for diseases characterized by aberrant TrxR1 expression. 

### 2.3. Linear Diselenide as the Trigger 

**Figure 8:**
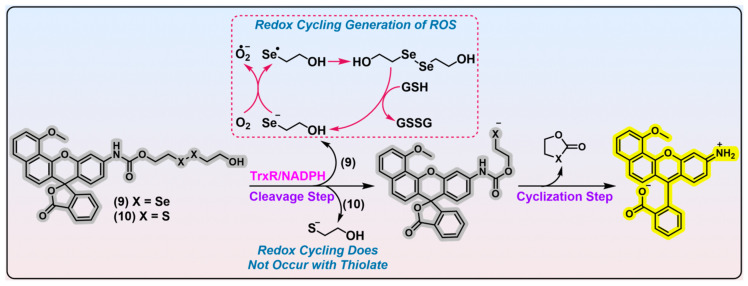
Linear diselenide-based TrxR probe. Probes 9 and 10 are diselenide- and disulfide-based TrxR probes. Experimental evidence indicates probe 9 is better than probe 10 in recognizing TrxR. During the activation process of probe 9 by TrxR, free selenolate is generated, which leads to the production of ROS. The dashed box in red shows the process of generation of ROS through free selenolate byproduct, and the arrows indicate the transformation of the substances. The curved arrows in black show the generated side products after activation by TrxR. Protein TrxR is shown in pink. The colors covering the chemical structures show their fluorescence colors after TrxR activation, and the gray indicates the fluorescence of the probes has been quenched.

In 2020, Strongin et al. reported an off–on fluorescent probe specific for mammalian TrxR (Figure 8, probe 9; λ_em_/λ_ex_ = 580/531 nm). Probe 9 consists of seminaphthorhodafluor as the fluorophore, linear diselenide as the recognition part, and carbamate as the linker unit [114]. Docking simulations and kinetic studies (*K*_m_ = 15.89 μM) both indicate a favorable binding affinity of probe 9 with TrxR, in comparison with its disulfide counterpart probe 10. LC-MS analysis confirmed the proposed releasing mechanism shown in Figure 8, and a further imaging application of probe 9 in HCC827 cells with TrxR overexpressed shows its potential in TrxR detection. Notwithstanding its purported selectivity for TrxR, the confidence in its specificity may be significantly undermined by the following essential issues. First, previous studies unraveled that a diselenide bond in strained geometry is sensitive to GSH challenge [53,55], and linear diselenide is kinetically labile to background monothiols in theory [54]. According to this present study, probe 9 has a merely ~5-fold selectivity for TrxR (0.12 units) over the cellular abundant GSH (1 mM). Moreover, the applied concentration of GSH is a little low compared with physiological concentrations (~5 mM; it is even higher in cancer cells). This result was obtained upon a 20 min treatment, the incubation time of which is too short when considering the plateau time of probe 9 is ~30 min. Second, the research design fails to consider several critical interfering species, namely U498C TrxR, Trx, GR, GPx, Sec, and NADPH. Neglecting these species poses a significant challenge to claiming the selectivity of probe 9 for TrxR, as they exhibit either an overlapping biological functionality with TrxR or reactivity towards linear diselenide. Hence, it is imperative to verify whether the activation of probe 9 would be elicited by these particular species. Other disadvantages including a limited fluorescence increment and short Stokes shift of probe 9 also plague its further application. In addition, it may be deemed more appropriate to designate probe 9 as a theranostic agent, rather than a fluorescent probe for TrxR, given the substantial cytotoxicity exhibited by probe 9 towards SK-MEL-5 (IC_50_ = 3.5 μM) and A375 (IC_50_ = 4.8 μM) melanoma cell lines. Prior studies have unveiled that compounds containing diselenide bonds exhibit superior cytotoxicity over their disulfide counterparts [54,55]. The remarkable cytotoxicity of probe 9 may be attributed to the generation of a free selenolate upon reaction with TrxR (Figure 8, shown in red dashed box), and the selenolate is amenable to redox cycling in the presence of reducing species. This process leads to a rapid accumulation of excess ROS, which induces oxidative stress and cell death. 

Recently, a benzoselenadiazole-based theranostic agent was developed by Lin et al. for cancer treatment and cancer cell tracing/imaging [122], and mitochondrial TrxR may play a role in triggering this agent. However, the intrinsically unstable benzoselenadiazole moiety is widely known to be sensitive to many biological species including GSH, Cys, selenol, H_2_Se, homocysteine, protein kinases, and others [123,124,125,126,127,128,129]. Thus, it is imperative that the theranostic agent contrived by Lin et al. should be subjected to further improvement and reevaluation to ascertain its selectivity towards TrxR2. 

### 2.4. α,β-Unsaturated Ketone Moieties as the Triggers for Labelling/Imaging Agents 

**Figure 9:**
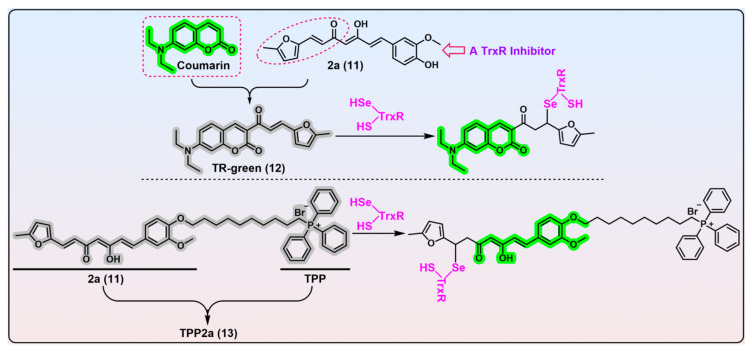
α,β-Unsaturated ketone-based TrxR probes. Probe 12, TR-green, is constructed from a specific TrxR inhibitor 2a (compound 11) and fluorescent coumarin. TR-green undergoes the Michael addition with the nucleophilic TrxR’s Sec residue. TR-green can be used in gel-imaging of TrxR and live-cell imaging of TrxR. Probe 13, TPP2a, is built by the integration of compound 2a with TPP. TPP2a can be used as a labelling agent for TrxR2, or a theranostic agent for cancers overexpressing TrxR2. The dashed box in red shows the structure of fluorophore coumarin, and the dashed ellipse in red show the structural determinant of compound 2a for inhibition of TrxR activity. Protein TrxR is shown in pink. The colors covering the chemical structures show their fluorescence colors after TrxR activation, and the gray indicates the fluorescence of the probes has been quenched.

Compounds containing α,β-unsaturated ketone moieties have long been documented as TrxR inhibitors, due to their potential to undergo the Michael addition with reactive Sec residue in the C-terminal active site of TrxR under physiological conditions [84,130,131,132,133,134,135,136,137,138,139,140]. Inspired by this interesting chemistry, the Bu group developed an α,β-unsaturated ketone-based fluorogenic probe for the covalent labelling of TrxR (Figure 9, TR-green, probe 12) [141]. Prior to this work, Bu et al. identified compound 2a (Figure 9, compound 11) as a highly selective TrxR inhibitor from a library of synthesized compounds and proved the furanyl–acryl moiety is the structural determinant responsible for its reactivity with TrxR [142,143]. Further, the conjugation of the fluorophore coumarin with the furanyl–acryl moiety as the recognition part generates the off–on probe TR-green (λ_em_/λ_ex_ = 500/440 nm). A biological evaluation revealed TR-green has great potential in the gel-imaging of TrxR, determining the concentrations of TrxR in the nanomolar level, and live-cell imaging of TrxR. 

Unlike the aforementioned probes that are activated under the catalytic function of TrxR, the current probe exhibits a notable limitation in that it engages with TrxR in a 1:1 stoichiometric ratio, thereby compromising TrxR’s enzymatic activity. On this ground, TR-green may be more preferred to be applied as a labelling agent or theranostic agent rather than a live cell-based imaging agent. Further, the TR-green suffers from a non-negligible deficiency insofar as the potential interference stemming from Sec remains undetermined. This shortcoming is of particular significance, in light of the trigger of TR-green as an α,β-unsaturated ketone motif. The sensitivity and selectivity of TR-green towards TrxR may benefit from further improvement, given that the concentrations of TrxR, Trx, and GSH utilized in this present study deviate a little from those that are physiologically relevant. 

Based on compound 2a (Figure 9, compound 11), Bu et al. further developed a mitochondria-targeted theranostic agent TPP2a (Figure 9, probe 13) [116]. Due to the presence of a triphenylphosphonium (TPP) unit in the structure, TPP2a manifests a potent TrxR2-targeting ability, which results in a remarkable burst of cellular ROS and subsequent oxidative stress-induced cell death via a mitochondrial pathway. Compared with parent compound 2a, the cytotoxicity of TPP2a is enhanced at least 10-fold against a variety of cancer cell lines, indicating the efficacy of a mitochondria-targeting approach. TPP2a has an environment-dependent fluorescence property (λ_em_/λ_ex_ = 500/440 nm) when it is located in a hydrophobic environment of TrxR2. Thus, interference from small molecules is insignificant; however, it is obvious when TPP2a is encountered with other proteins that have hydrophobic pockets suitable for TPP2a. To avoid this non-specific fluorescence, the authors utilized a coordination strategy to promote TPP2a’s labelling and imaging selectivity for TrxR2 in cells. Cupric ion was found to quench the fluorescence of TPP2a, through coordination with the β-diketone motif of TPP2a leading to a broken conjugation system, whereas TPP2a may have a superior binding affinity for TrxR2, arising from two possible hydrogen bonds formed between the K29 residue in the N-terminus of TrxR2 and β-diketone motif of TPP2a. This helps TrxR2 to reverse the fluorescence of coordinated TPP2a; however, other proteins cannot. The phenomenon was then rationalized by docking studies. The elegant cupric ion-mediated coordination strategy leverages the preferential binding of TPP2a with TrxR2 to circumvent the potential interference from other functionally or structurally relative proteins, thereby fulfilling the selective imaging and labelling of TrxR2 in cells. 

### 2.5. Linear Disulfide as the Trigger 

**Figure 10:**
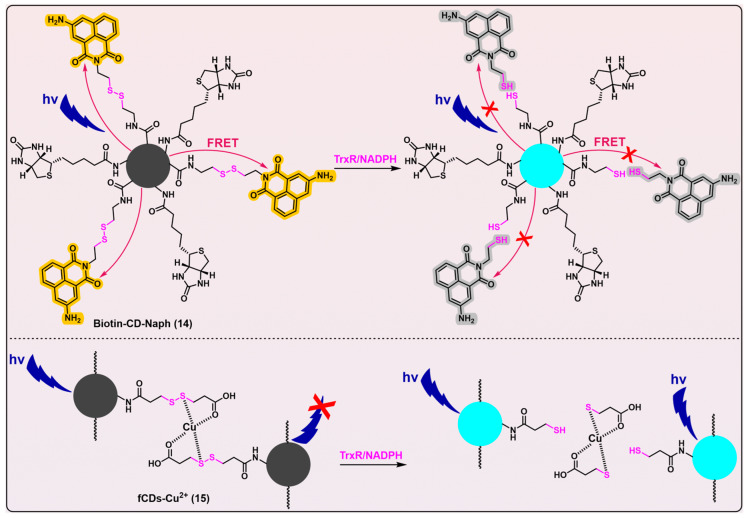
Linear disulfide-based TrxR probes. Probes 14 and 15 are based on carbon dots, and both have linear disulfide as their recognition parts for TrxR. Biotin-CD-Naph is a ratiometric probe that uses biotin to selectively target cancer cells. When the disulfide bond in Biotin-CD-Naph is cleaved by TrxR, the FRET process is blocked, and carbon dots emit fluorescence. fCDs-Cu^2+^ is quenched because of the chelation of Cu^2+^ within this probe. When the disulfide bond in fCDs-Cu^2+^ is cleaved by TrxR, Cu^2+^ is removed from the surface of carbon dots, and the fluorescence generates. Both probes 14 and 15 are able to image cancer cells that overexpress TrxR. The curved arrows in red indicate the FRET process, which leads to the turning-on of naphthalimide fluorophore (shown in yellow) and quench of carbon dots (shown in grey). The red **×** in the upper panel indicates the blockage of FRET process, which results in turning-on of carbon dots (shown in blue) and quench of naphthalimide fluorophore (shown in grey). The red **×** in the bottom panel indicates the quench process by chelation of Cu^2+^. Protein TrxR is shown in pink.

Compared with traditional imaging agents, carbon dots have several advantages such as good aqueous solubility, wide pH tolerance, high photostability, and easy surface functionalization. Thus, fluorescent probes based on carbon dots have been finding various applications in recent years [144,145]. Singh et al. developed two carbon dots-based analytical tools for detecting mammalian TrxR (Figure 10, probes 14 [146] and 15 [147]). Both Biotin-CD-Naph (λ_em_/λ_ex_ = 450/360 nm) and fCDs-Cu^2+^ (λ_em_/λ_ex_ = 446/340 nm) have linear disulfides as their triggers, that are designed to be cleaved by TrxR. Biotin-CD-Naph is a ratiometric probe, constructed on the basis of a fluorescence resonance energy transfer (FRET) mechanism. Whilst Biotin-CD-Naph shows a discernible level of TrxR selectivity and demonstrates potential applicability in cancer cell imaging and screening, the specificity of the probe still needs refinement because the employment of a disulfide bond as the trigger likely makes the probe susceptible to other disulfide-reducing species, e.g., Trx. Fortunately, Biotin-CD-Naph was additionally attached with biotin as a supplementation moiety, thereby enabling the preferential and selective recruitment and imaging of cancer cells that overexpress biotin receptors such as avidin and streptavidin on their cellular surfaces. Given the potential cytotoxic effects of 3-aminonaphthalimide, as well as the ROS-generating capabilities of carbon dots under photic conditions [148,149,150], Biotin-CD-Naph holds immense promise to be applied as a theranostic agent in cancer treatment. 

Another carbon dots probe fCDs-Cu^2+^ (Figure 10, probe 15) has been developed as an off–on fluorogenic type, with demonstrated selectivity for TrxR over a series of other biological species. Additionally, this probe has been successfully utilized for the imaging of cancer cells [147]. However, the same problem suffered by Biotin-CD-Naph (Figure 10, probe 14) also pertains to fCDs-Cu^2+^, i.e., a possible insufficient specificity of disulfide bond in fCDs-Cu^2+^ for TrxR. Thus, the potential lack of selectivity of Biotin-CD-Naph and fCDs-Cu^2+^ towards TrxR underscores the pressing need for significant enhancements in their specificity. 

Drug delivery systems based on disulfide bond usually target reducing the environment in cells. However, conferring individualized selectivity upon a given target through the utilization of the disulfide moiety presents a formidable challenge, given the presence of multiple cellular disulfide-reducing enzymes and a pronounced background of monothiols that exhibit different degrees of potential for the irreversible cleavage of the moiety. Perhaps exploiting the kinetic differences in various disulfide compounds towards TrxR and other biological species may contribute to the solution of this intractable selectivity [115]. 

## 3. Conclusions and Perspectives 

The Trx system is one of the principal networks in cellular redox biology, and it executes multitudes of biological functions mainly through the reduced Trx via thiol–disulfide exchange reactions [3,4]. Importantly, cellular Trx is primarily maintained in its reduced state through the enzymatic activity of TrxR, although alternative pathways involving Grx2 and GSH can also contribute to the reduction of oxidized Trx in cells genetically deprived of TrxR, due to substantial interconnections within the cellular redox network [22,151,152,153,154,155,156]. Thus, TrxR orchestrates a pivotal role in preserving cellular redox homeostasis and supporting a wide range of signaling pathways that regulate diverse physiological processes, including but not limited to cell proliferation, differentiation, death, and numerous other crucial events [21,27,28]. The dysregulation of TrxR has been cumulatively unraveled to be implicated in an increasing number of diseases, such as cancer, neurodegenerative diseases, and autoimmune/inflammatory diseases, among others. Consequently, targeting the modulation of TrxR has gradually emerged as a promising therapeutic strategy for various pathological conditions in recent years [1,2,6,29,31,36,37,38,157]. 

Owing to the paramount importance of TrxR in both physiological and pathological processes, it is imperative to develop reliable methods for monitoring its dynamics in live cells, as this will largely enhance our deep appreciation of the multifaceted functions of TrxR in redox biology. Until now, several classic assays have been well-established and widely used for the determination of TrxR activity [39,40]. However, conducting these assays typically requires either purified TrxR or lysed cells, which faces technical and cost-related challenges [158]. Moreover, these assays fail to give real-time information on the fluctuation of TrxR activity in live systems. Several imaging techniques have partially conquered these drawbacks, among which molecular fluorescent probes-based imaging and quantifying analysis show great promise in dynamically providing informative data of desired proteins in their native environments [69]. 

Fluorescent probes generally offer high sensitivity and specificity, as well as non-invasive and rapid analysis, making them advantageous over other techniques and assays for investigating cellular processes in complex matrixes [64,65,66]. Moreover, the spectroscopic properties of fluorescent probes can be readily tuned because of their structural tailorability, which can improve their spatial resolution, sensitivity, binding affinity to target proteins, and other related characteristics. However, designing high-quality fluorescent probes with superior specificity for target proteins remains a considerable challenge, as the mutual interaction between a protein and its native substrates is generally more potent and specific than that of proteins with probes in the heterogeneous cellular milieu [69]. As for the precise quantification and imaging of TrxR in cells, a probe should selectively discriminate TrxR from manifolds of proteins or biomolecules that perform similar chemistries, such as Trx, GR, Grx, GSH, TRP14, and others. Luckily, mammalian TrxR possesses unique structural features that are absent from other functionally/structurally relevant enzymes. Specifically, the conserved C-terminal elongation of TrxR contains a unique active site equipped with a selenolthiol pair. This property endows TrxR with elevated reaction kinetics and lowered reduction potential, which can be intentionally exploited for the design of selective probes with higher reaction kinetics towards TrxR and/or lower reduction potential than other interfering biological species. Obviously, achieving specific binding with the C-terminal active site of TrxR may pose a considerable challenge, as the active site of TrxR is structurally flexible and exposed on the surface of the protein. This renders TrxR not easily amenable to weak mutual interaction with potential fluorescent probes. However, several previous cases have shown some potential in this regard [89,114,116,159,160,161]. In order to provide readers with a succinct and efficient comparative analysis of the present TrxR probes, Table 1 presents a comprehensive summary of these probes. 

### 3.1. Can 1,2-Dithiolane Be Employed as the Trigger for TrxR Probes? 

Based on the reaction mechanism of TrxR, with its wide spectrum of native substrates, a series of activity-based probes were reported in the past few years. Accordingly, several types of recognition parts were employed to construct novel TrxR fluorescent probes and prodrugs.

On one hand, 1,2-dithiolane has the reduction potential of −240~−270 mV which is slightly lower than that of GSH (~−240 mV), a major cellular interfering species for TrxR probes [78,80,119]. Moreover, Trx (~−230 mV) [162] and Grx (~−200 mV) [163] cannot reduce 1,2-dithiolane in thermodynamics, whereas TrxR (~−300 mV) [164,165] can. This constitutes the thermodynamic basis for the TrxR-mediated reduction of 1,2-dithiolane probes. Further, the unique structural features of TrxR confer kinetic advantages that promote its reaction with 1,2-dithiolane probes. Hence, several 1,2-dithiolane-based fluorescent probes and prodrugs were developed and shown to be selective in targeting TrxR [166,167]. 

On the other hand, 1,2-dithiolane, a strained cyclic moiety featuring a dihedral angle of ~30°, exhibits high energy in comparison with unstrained linear disulfides (~90°), rendering it highly reactive towards numerous biological thiols in terms of kinetics [168,169,170,171,172,173,174]. Interestingly, natural compounds containing diketopiperazine structures with a dihedral angle of ~0° demonstrate non-discriminatory interactions with biological targets, which can be attributed to the presence of highly strained disulfide bonds within their structures. Consequently, the reduction-induced opening of 1,2-dithiolane is an irreversible process due to the unfavorable energetics associated with the reclosure of its disulfide bond. This irreversible opening can occur through exposure to high concentrations of background monothiols and thiol-mediated cellular uptake facilitated by cytomembranes [56,168,174,175,176,177,178,179,180,181,182]. Additionally, there exists the possibility of 1,2-dithiolane probes undergoing a reduction-independent reaction, known as strain-promoted *r*ing-*o*pening *p*olymerization (ROP), which is contingent upon the concentration of the probe [80,169,174,183,184,185,186]. Thus far, the currently available 1,2-dithiolane probes, such as TRFS series probes, have been shown to lack specificity towards TrxR [80]. These observed effects and results collectively highlight the non-specific nature of 1,2-dithiolane with regards to TrxR, or any other dithiol enzymes, thereby presenting a challenge in its application as a selective trigger for TrxR probes. Nonetheless, it is noteworthy that 1,2-dithiolane probes incorporating distinct fluorophores and linkers exhibit significantly disparate fluorogenic properties, implying that chemical modifications can engender substantial variations in the characteristics of 1,2-dithiolane [53]. While the inherent structure of 1,2-dithiolane alone exhibits limited specificity, the potential for selectivity can be enhanced by adjusting the cargoes and linkers appended to 1,2-dithiolane or through direct chemical modifications of the moiety. Hence, comprehensive endeavors are still imperative to delve deeper into the biochemical facets of 1,2-dithiolane at multiple levels, with the prospect that novel derivatives may unexpectedly emerge and demonstrate definitive specificity for TrxR. 

### 3.2. How Can We Achieve Selectivity towards TrxR Using a 1,2-Dithiane Scaffold? 

**Figure 11:**
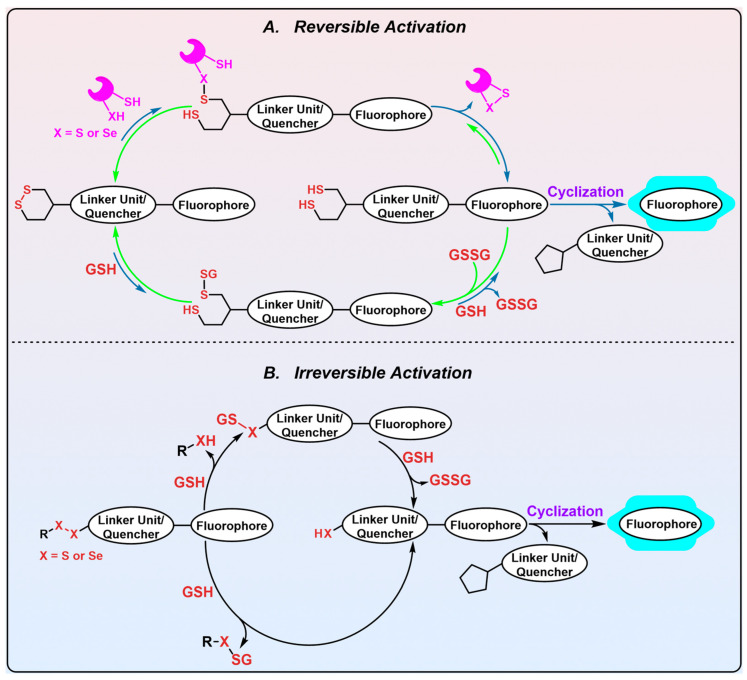
Possible activation pathways and mechanisms of linear disulfide/diselenide and 1,2-dithianes by monothiols and dithiol/selenolthiol enzymes. (**A**). The bidirectional arrows indicate the transformation is reversible. The blue arrows indicate the forward processes, while the green arrows show the reverse processes. The pink icons having dithiol/selenolthiol on them represent enzymes in biological systems. (**B**). The one-way arrows in black show the irreversible activation processes of the probe.

According to the literature, 1,2-dithianes have been observed to exhibit a resistance to opening by monothiols and TrxR [53,79,119]. This property may be attributed to the pathways in which 1,2-dithianes can undergo a reversible thiol–disulfide exchange reaction when attacked by monothiols or dithiol proteins (Figure 11A). Furthermore, the reduction potential of 1,2-dithianes (−317~−327 mV) is significantly lower than that of TrxR and GSH [187,188,189]. These effects may contribute to the general stability of six-membered cyclic disulfide compounds. 

Interestingly, it was recently reported that stereoisomeric monocyclic and annelated bicyclic 1,2-dithiane scaffolds exhibit selectivity towards the dithiol protein Trx, indicating the tremendous potential to harness and modify the chemical structures and conformations of 1,2-dithianes to achieve the desired specificity [119,121]. Regarding the selectivity for TrxR, it is rational to consider augmenting the electrophilic nature of 1,2-dithianes to further enhance the reaction kinetics between TrxR and 1,2-dithianes, given the significant reaction kinetics of TrxR. Utilizing the mutually advantageous kinetics, 1,2-dithianes possess the potential to selectively target TrxR and simultaneously offset the potential risk of thiol–disulfide exchange reactions caused by high concentrations of monothiols or other dithiol proteins. Thorn-Seshold et al. have demonstrated that replacing one of the sulfur atoms in 1,2-dithianes with a selenium atom results in the formation of a cyclic selenenylsulfide scaffold called 1,2-thiaselenane, which has great potential to address the issue of selectivity [79]. The presence of selenium in 1,2-thiaselenane significantly contributes to its enhanced reaction kinetics, exhibiting a rate that is approximately 10^4^-fold faster when compared with that of 1,2-dithianes. Moreover, this interaction affords the formation of thermodynamically favorable intermolecular diselenide intermediates. Then, the intermolecular diselenide intermediate can be readily resolved by the neighboring Cys497 residue present in the C-terminal active site of TrxR, leading to the formation of a fully reduced selenolthiol form of 1,2-thiaselenane and an oxidized form of TrxR. 

Although selenium replacement also increases the initial reaction kinetics of the first molecule of GSH with 1,2-thiaselenane, resolving the nascent intermolecular selenenylsulfide bond with the second molecule of GSH is kinetically and thermodynamically unfavorable. Hence, selenium replacement in 1,2-dithianes not only recruits the desired TrxR, but also blocks interfering background monothiols attack. However, it should be noted that the elevated reaction kinetics of 1,2-thiaselenane may also increase its reactivity with dithiol proteins, potentially compromising its specificity for TrxR. To address this issue, novel recognition parts based on the 1,2-thiaselenane scaffold could be designed with structural elements that lead to lower or at least equivalent reduction potential compared with dithiol proteins, thereby improving selectivity for TrxR. It is of particular importance to pay attention to dithiol proteins that have a small number of biologically native downstream substrates or comparable reduction potential to TrxR. These dithiol proteins may present challenges to the selective recognition of TrxR by 1,2-thiaselenane-based probes. Further innovative improvement of 1,2-thiaselenane as an exceedingly selective substrate for TrxR would pave an avenue for the development of various high-quality TrxR-triggered fluorescent probes, prodrugs, and theranostic agents. 

### 3.3. Other Chemical Candidates as Selective Substrates for TrxR? 

Linear disulfide/diselenide compounds are generally considered non-selective towards any enzymes, as they are irreversibly cleaved by various biological thiols (Figure 11B) [190,191]. In particular, diselenide compounds exhibit kinetically superior reactivity towards thiols, thereby further diminishing their enzyme specificity. To attain selectivity of these types of triggers for a desired enzyme, it is possible to enhance the reaction kinetics of the triggers with the target protein relative to other interfering species [115,192,193,194,195]. 

It is noteworthy that diselenide-based probes should be developed and applicable as theranostic agents rather than fluorescent probes for TrxR, since the free selenols generated by the reduction of diselenide compounds are prone to redox cycling (Figure 8). This leads to the rapid production and accumulation of ROS and subsequent oxidative stress-induced cell death [54,55,114]. A similar outcome is likely to occur with α,β-unsaturated ketone-type TrxR probes, due to the formation of SecTRAPs (selenium compromised thioredoxin reductase-derived apoptotic proteins) through the covalent reaction of the probes with the Sec residue in the active site of TrxR [1,61,196,197,198,199]. While fluorescent imaging of TrxR is fulfilled, the enzyme is inactivated by using α,β-unsaturated ketone-type TrxR probes. However, this does not restrain this type of probe from being legitimately applied as a potential labelling/imaging agent. 

### 3.4. Potential Approaches for the Development of High-Quality TrxR Probes 

As previously described, the available recognition units and sensing mechanisms that enable the selective targeting of TrxR are limited. Therefore, supplementary methodologies can be employed to expedite the discovery of novel TrxR probes. For example, computer-aided molecular docking, as was performed by Strongin et al. [114] and Bu et al. [116], can be utilized to guide the rational design of selective TrxR probes or improve the response rate of TrxR probes. Additionally, the use of inhibitor bionics proved to be advantageous in identifying novel recognition parts for TrxR probes [100,200]. The utilization of these methods may facilitate the development of TrxR probes that exhibit the selective identification of individual isoforms within the TrxR1-3 family of enzymes. Recently, highly selective inhibitors of TrxR were identified [81,82], which may enlighten the construction of selective TrxR probes. 

Deep learning of novel sensing mechanisms may be an instrumental strategy for the development of highly selective probes for TrxR, just as in the way of discovering Fast-TRFS [53]. Inasmuch as TrxR has superior reaction kinetics over other structurally similar and functionally overlapping biological species, the untapped ultrafast sensing mechanism may help largely to reduce interference from these species in a short length of time.

Up to now, there has been no fluorescent probe that can be used for the intravital real-time imaging and activity detection of TrxR. The short-wavelength emission TrxR probes that are currently in common use lack the capability to acquire accurate and real-time information of TrxR within live organisms, primarily due to the limited depth of tissue penetration. Therefore, the development of long-wavelength emission TrxR probes is currently urgently needed. Considering the critical involvement of TrxR in tumorigenesis, the *n*ear-*i*nfra*r*ed (NIR) TrxR probes exhibit great potential for directing intraoperative navigation in tumor resection [201]. In order to enhance the resolution and accuracy of in vivo imaging using NIR probes for TrxR, various techniques such as photoacoustic imaging and magnetic resonance imaging can be integrated to develop multifunctional fluorescent probes that could potentially improve the diagnosis of early-stage cancer and assist in interventional surgical imaging in complicated biological environments.

### 3.5. Issues in the Development of TrxR Probes 

An important matter that arises when assessing or verifying the selectivity of TrxR probes pertains to the auranofin (AF) assay. Currently, AF is an oral antirheumatic drug that is clinically administered, and it is also commonly employed as a TrxR inhibitor in diverse biological assays [23,43,44,202,203]. TrxR has been identified as a major target of AF, albeit it is worth noting that more than twenty proteins, in addition to TrxR, are subject to covalent inhibition by AF. Despite extensive research efforts, the precise therapeutic mechanism(s) of action of this compound remain elusive [45,46,204,205]. As a lipophilic transition metal-based coordination compound, AF has strong reaction tendency with chalcogen compounds [43,44,206,207,208,209]. First, considering the chemical component features of TrxR’s substrates, it is necessary to determine whether the developed TrxR probes are reactive with AF. Second, it is highly recommended to employ metal-free TrxR inhibitors instead, e.g., Tris [81,82], in conducting competition assays to confirm the selectivity for TrxR. Third, an additional vital aspect to consider when evaluating the selectivity of TrxR probes is to perform orthogonal measurements, as exemplified in the development of the selective TrxR1 probe RX1 [79]. These may include cell-free titrations of reducing species/enzymes, the cellular knockout/knockdown/knock-in of TrxR, and the activity modulation of TrxR using chemical approaches. Such cross-validation measures are necessary to achieve a comprehensive assessment of the selectivity of probes [80,119,121,210,211,212]. 

Selectivity is a stringently condition-dependent phenomenon that is contingent upon various factors, including incubation time, analyte concentration, and testing matrix. This complexity is further compounded and exacerbated when the selectivity of a probe is assessed in a biological milieu. Furthermore, the selectivity of a probe can also be influenced by the physicochemical properties of the analyte and the probe itself, such as their size, charge, and hydrophobicity. It is important to recognize that selectivity is not an absolute property of a probe, but rather a relative measure of its ability to discriminate against other biological species present in the sample matrix. While achieving perfect selectivity is challenging among the fluorescent probes developed for TrxR1 assessment, Thorn-Seshold et al.’s RX1 probe has emerged as the most superior option to date. Finally, it is worth noting that the development of novel and improved TrxR probes with enhanced selectivity, sensitivity, and multifunctionality remains an active area of research, driven by the escalating demand for the reliable and accurate detection and quantification of cellular redox status. We anticipate this review will offer valuable insights into the future development of TrxR fluorescent probes, prodrugs, and theranostic agents. We also believe TrxR probes with improved properties are bound to upsurge in the foreseeable future, which are expected to contribute towards a better understanding of TrxR’s functions in redox biology and the development of effective diagnostic and therapeutic tools for diseases that are intimately associated with TrxR. 

## Figures and Tables

**Table 1:** Properties of the current TrxR probes.

Probe No. and Original Name	Recognition Moiety	λ_em_/λ_ex_ (nm)	*K*_m_	Response Rate (min)	LOD (nM)	Application	Ref.
(1) TRFS-green	1,2-dithiolane	538/438	Other ^1^	~240	—	Live cells	[78]
(2) TRFS-red	1,2-dithiolane	660/615	Other ^2^	~120	—	Live cells	[101]
(3) TP-TRFS	1,2-dithiolane	490/370	—	~180	—	Live cells and animals	[102]
(4) Mito-TRFS	1,2-dithiolane	540/438	—	~60	—	Live cells	[110]
(5) DSMP	1,2-dithiolane	510/340	12.5 μM	~12	—	Live cells and animals	[112]
(6) Fast-TRFS	1,2-dithiolane	460/345	—	~5	—	Live cells	[53]
(8) RX1	1,2-thiaselenane	520/355	—	~180	—	Live cells	[79]
(9) 1a	Linear diselenide	580/531	15.89 μM	~30	—	Live cells	[114]
(12) TR-green	α,β-unsaturated ketone	500/440	—	~30	—	Live cells	[141]
(13) TPP2a	α,β-unsaturated ketone	500/440	—	~30	—	Live cells	[116]
(14) Biotin-CD-Naph	Linear disulfide	450/360	7 μg	~80	72	Live cells	[146]
(15) fCDs-Cu^2+^	Linear disulfide	446/340	5.5 μg	~100	20	Live cells	[147]

^1^ 63.2 μM/0.11 s^−1^ as the *K*_m_/*k*_cat_ value. ^2^ 51.8 μM/0.17 s^−1^ as the *K*_m_/*k*_cat_ value.

## Data Availability

Not applicable.
